# The serum gamma-glutamyl transpeptidase-to-platelet ratio predicts HELLP syndrome

**DOI:** 10.1186/s12884-025-07431-4

**Published:** 2025-03-15

**Authors:** Jiaying Chen, Hao Gu, Hongqin Wu, Minhui Jiang, Ying Gu, Yaling Feng

**Affiliations:** 1 Department of Women Health Care, Wuxi School of Medicine, Wuxi Maternal and Child Health Hospital, Jiangnan University, Wuxi, 214002 Jiangsu Province PR China; 2 Department of Obstetrics, Wuxi School of Medicine, Wuxi Maternal and Child Health Hospital, Jiangnan University, Wuxi, 214002 Jiangsu Province PR China

**Keywords:** HELLP syndrome, Gamma-glutamyl transferase to platelet ratio, Predictions, Adverse pregnancy outcomes

## Abstract

**Background:**

HELLP (Hemolysis, elevated liver enzymes, and low platelets) syndrome is a dangerous obstetric condition that is in great need of simple and inexpensive non-invasive early predictors, but it has been poorly studied. This study was conducted to investigate the predictive role of serum gamma-glutamyl transpeptidase to platelet ratio (GPR) during pregnancy in HELLP syndrome and its adverse pregnancy outcomes.

**Methods:**

This was a retrospective study in a tertiary hospital. One hundred parturients were allocated into two groups: HELLP group (*n* = 50) and control group (*n* = 50).

**Results:**

① In the HELLP group, the maternal GPR levels showed a continuous upward trend from middle pregnancy to before-delivery, with significantly higher values observed in late pregnancy and before-delivery compared to the control group (*P* < 0.05). ② A comparison was made between the counts of platelets (PLT), plasma fibrinogen (FIB), alanine transaminase (ALT), aspartate transaminase (AST), uric acid (UA), γ-glutamyl transferase (GGT), and GPR in two groups of the pregnant women during their late pregnancy and before-delivery to the hospital, all of which showed statistically significant differences (*P* < 0.05). ③Multivariate logistic regression analysis showed that higher GPR, ALT, and UA were independent risk factors for the development of HELLP syndrome (OR = 23.382, 1.169,1.016, *P* < 0.05), while higher FIB was a protective factor (OR = 0.057, *P* < 0.05). ④ Spearman correlation analysis indicated that the abnormal elevation of GPR in late pregnancy and before-delivery was correlated with preterm birth (*r* = 0.510, 0.450, *P* < 0.05). ⑤ROC curve analysis revealed that the predictive efficacy of GPR in late pregnancy (AUC = 0.8441) was higher than AST (AUC = 0.7960), ALT (AUC = 0.7952), and PLT (AUC = 0.7691) in late pregnancy, with an AUC of 0.8656 for GPR before delivery When GPR values were 0.22 and 0.27 in late pregnancy and before-delivery, the sensitivity for predicting HELLP syndrome was 77.6% and 78%, and the specificity was 85% and 90%.

**Conclusions:**

The abnormal increase of GPR during pregnancy has a certain predictive effect on HELLP syndrome and its adverse pregnancy outcomes.

## Background

HELLP syndrome (Hemolysis, elevated liver enzymes, and low platelets syndrome) is a rare and severe complication of hypertension in pregnancy, mainly manifested as hemolysis, elevated liver enzymes, thrombocytopenia, which can lead to maternal and perinatal mortality [1]. The risk of developing HELLP syndrome during pregnancy is about 0.5–0.9%, but it rises dramatically to 10–20% in pregnant women with pre-eclampsia [2]. The majority of cases of HELLP syndrome happen in late pregnancy (28 to 40 weeks). Due to the disease’s rapid progression, if it is not detected and treated in time, the pregnant women may experience serious complications, such as placental abruption, disseminated intravascular coagulation, acute kidney injury, liver capsule rupture and other serious complications. Infants in the perinatal period are at increased risk for respiratory distress syndrome, premature birth, and higher mortality rates [3]. Therefore, discovering effective and simple predictive indications is vital for the early detection and treatment of HELLP syndrome. Lemoine et al. [4] first proposed that the gamma-glutamyl transferase to platelet ratio (GPR) as a novel inflammatory indicator for predicting the risk of liver fibrosis and cirrhosis in patients with chronic hepatitis B virus. Currently, there is no relevant research exploring whether GPR can predict HELLP syndrome. Therefore, our study intends to evaluate the predictive significance of GPR in HELLP syndrome and its adverse consequences for pregnancy. It will provide innovative ideas and evidence for early identification and recognition of HELLP syndrome in clinical practice, improving pregnancy outcomes.

## Materials and methods

### Patients

This retrospective study included 100 women admitted to our hospital from January 2017 to June 2024. They were divided into two groups of 50 people, HELLP syndrome group and control group. Among the 50 HELLP syndrome patients, 28 were diagnosed with antepartum and 22 postpartum. To control for the potential confounding factor of delivery mode, we selected only cesarean section patients for the study. The inclusion criteria for the control group were healthy pregnant women who underwent cesarean sections due to breech presentation or cephalopelvic disproportion. Patients with HELLP syndrome should fulfill the American Tennessee criteria [5]: (1) Hemolysis, defined by abnormal peripheral smear, increased bilirubin (≥ 20.5 µmol/L) and increased lactic dehydrogenase(≥ 600 U/L). (2) Elevated Liver enzymes, defined as ALT ≥ 40 U/L or AST ≥ 70 U/L. (3) Low Platelets, defined as platelet (PLT) count < 100 × 10/L. Exclusion criteria: Cases those had pregnancy with chronic hypertension, gestational diabetes, intrahepatic cholestasis of pregnancy, hepatitis, lupus or other immune disorders and primary immune thrombocytopenia. The study protocol was approved by the ethics committee of our institution (Registration number: 2024-06-0507-13). The study was conducted following the tenets of the declaration of Helsinki. Before conducting this study, we obtained their informed consent through telephone return visits.

### Indicators of observation

A retrospective case-control study was conducted to collect the general clinical data, some laboratory indexes during pregnancy and adverse pregnancy outcomes in two groups of pregnant women. (1) General clinical data: Age, Body Mass Index (BMI), number of pregnancies, number of births, gestational age at birth, systolic blood pressure (SBP), diastolic blood pressure (DBP), mean arterial pressure (MAP), adverse pregnancy history (including unexplained spontaneous abortion, multiple induced abortions, embryo arrest, congenital disabilities, etc.), history of hypertension during pregnancy, and family history of hypertension. (2) Laboratory indexes include total bilirubin (TBil), creatinine (Cr), uric acid (UA), fibrinogen (FIB), activated partial thromboplastin time (APTT), hemoglobin (HGB), platelets (PLT), alanine aminotransferase (ALT), aspartate aminotransferase (AST), and gamma-glutamyl transferase (GGT). GPR is derived from GGT and PLT using the following formula: GPR = (GGT/upper limit of normal GGT)/PLT*100, during which the upper limit of normal GGT is 35 U/L [6]. (3) Adverse pregnancy outcomes include low birth weight, stillbirth, early delivery, fetal distress, and admission of the mother to the Maternal Intensive Care Unit (MICU).(4)The early pregnancy period spans from the first week to the 13th week and 6 days of gestation (less than 14 weeks). The mid-pregnancy period extends from the 14th week to the 27th week and 6 days (14 to 28 weeks). The late pregnancy period begins at the 28th week and continues until delivery.

### Statistical analysis

Statistical analysis was performed using SPSS version 27.0. The measurement data were tested by Kolmogorov-Smirnov normality test and the mean ± standard deviation (± S) (normal distribution data) or median (P25, P75) (skewed distribution data) was used for statistical description. The independent sample t-test (normally distributed data) or Mann-Whitney U test (skewed data) was used to compare group differences. Count data are expressed as cases (%), and the difference between groups is tested using the chi-square or Fisher’s exact test. Integration of statistically significant indicators in single-factor analysis into multi-factor logistic regression analysis, after adjusting for co-linearity, the risk factors for the onset of HELLP syndrome were analyzed. The results are presented as odds ratios (OR) with 95% confidence intervals (CI). Spearman correlation analysis was utilized to investigate the correlation between GPR and adverse pregnancy outcomes. The receiver operating characteristic (ROC) curve was drawn to assess the predictive value of GPR for HELLP syndrome and obtain the best cut-off value. A probability of ≤ 0.05 was considered to be statistically significant.

## Results

### General clinical data comparison

Compared with the control group, the differences in BMI, pregnancy, number of deliveries, gestational age of delivery, SBP, DBP, MAP and adverse pregnancy history of pregnant women in the HELLP group were statistically significant (*P* < 0.05). There was no significant difference in age, history of hypertension during pregnancy and family history of hypertension between the two groups (*P* < 0.05) (Table 1).

**Table 1:** General clinical data of the two groups of pregnant women (*n* = 100)

Variables	HELLP group (*n* = 50)	Control group (*n* = 50)	*P* value
Age, years	30.55 ± 4.91	29.21 ± 3.24	0.122
BMI	28.37 ± 4.04	25.59 ± 3.44	< 0.001
Number of pregnancies	2(1,3)	1(1,2)	< 0.001
Number of previous births	0(0,1)	0(0,0)	< 0.001
Gestational age at delivery, weeks	33.93 ± 3.67	39.37 ± 1.08	< 0.001
SBP(mmHg)	152.67 ± 18.06	116.92 ± 8.87	< 0.001
DBP(mmHg)	97.82 ± 11.36	72.00 ± 6.53	< 0.001
MAP(mmHg)	117.38 ± 15.71	86.97 ± 6.42	< 0.001
History of adverse pregnancy and delivery (n, %)	13(26%)	4(8%)	0.03
History of gestational hypertension (n, %)	5(10%)	0(0)	0.056
Family history of hypertension (n, %)	7(14%)	3(6%)	0.182

### Comparison of PLT, GGT, and GPR in two groups of pregnant women

**Fig. 1:**
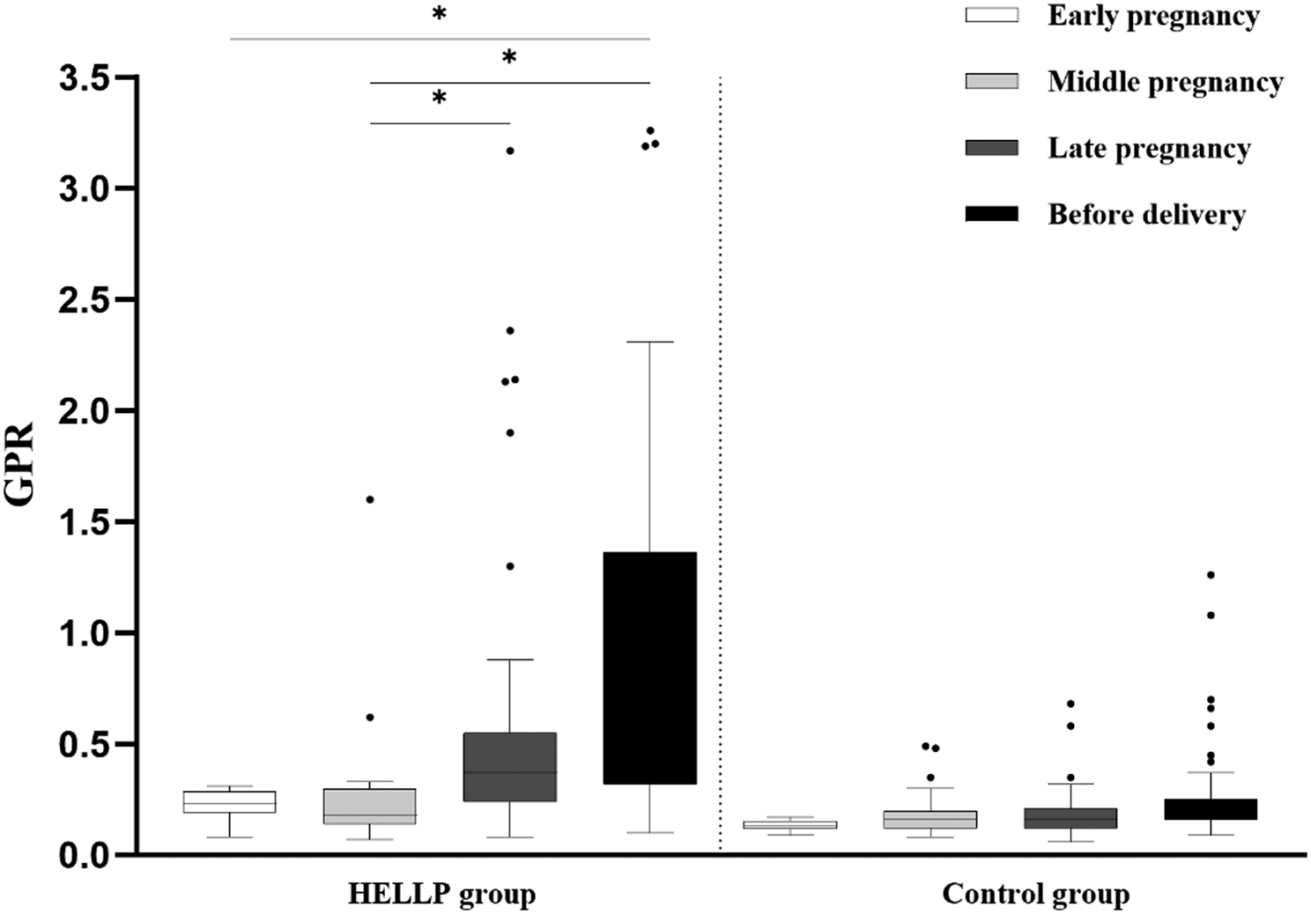
The trend of GPR changes in pregnant women in the HELLP group and the control group throughout pregnancy. The GPR of pregnant women in the HELLP group increased from mid-pregnancy to before-delivery and was significantly higher in the late-pregnancy and before-delivery than that in the mid-pregnancy. The GPR of pregnant women in the control group did not change significantly throughout pregnancy. *P < 0.05

**Fig. 2:**
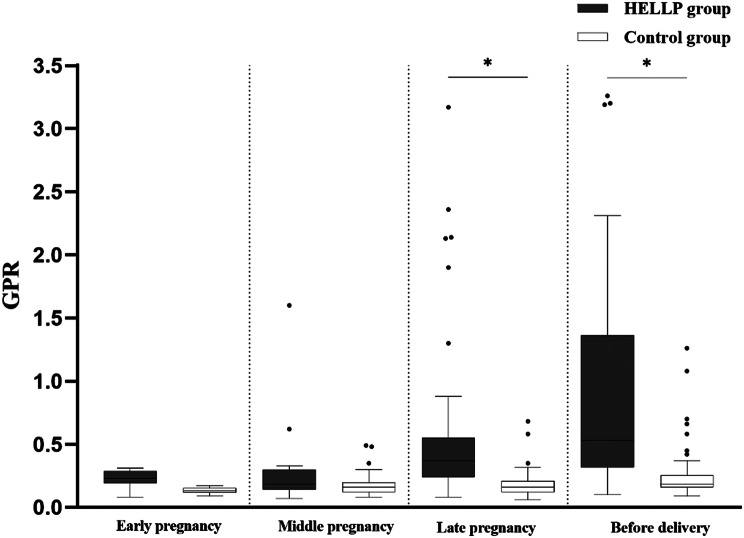
Compare the GPR of pregnant women in the HELLP group with that of the control group at different stages of pregnancy. The GPR of pregnant women in the HELLP group was significantly higher than that of the control group in the late-pregnancy and before-delivery. *P < 0.05

The PLT of pregnant women in the HELLP syndrome group showed a gradual downward trend during pregnancy, and was significantly lower than that of the control group in mid-pregnancy, late-pregnancy and before-delivery. GGT levels showed an upward trend from mid-pregnancy to before-delivery, and were significantly higher than that of the control group in the late-pregnancy and before-delivery, and the differences were statistically significant. (*P* < 0.05) (Table 2). In the HELLP group, GPR steadily increased throughout pregnancy, reaching significantly higher levels in late pregnancy and before delivery compared to mid-pregnancy (*P* < 0.05). However, the GPR of the control group remained stable throughout the pregnancy (*P* > 0.05) (Fig. 1). The GPR of pregnant women in the HELLP group in late-pregnancy and before-delivery was significantly higher than that of the control group, and the difference was statistically significant (*P* < 0.05) (Fig. 2).

**Table 2:** Analysis of PLT, GGT, and GPR in two groups of pregnant women (*n* = 100)

Variables	HELLP group (*n* = 50)	Control group (*n* = 50)	*P* value
PLT(×10^9^/L)			
Early pregnancy	223.42 ± 54.37	235.63 ± 36.50	0.316
Middle pregnancy	200.74 ± 53.78	222.12 ± 38.13	0.032
Late pregnancy	151.49 ± 54.75	212.43 ± 42.24	< 0.001
Before delivery	117.10 ± 49.54	190.36 ± 45.50	< 0.001
GGT(U/L)			
Early pregnancy	15.65(13.60,18.80)	10.75(9.65,11.63)	0.025
Middle pregnancy	12.10(9.20,16.40)	12.00(10.10,17.00)	0.625
Late pregnancy	20.00(13.00,31.50)	11.30(9.00,15.10)	< 0.001
Before delivery	24.40(13.15,48.03)	12.35(9.80,17.03)	< 0.001
GPR			
Early pregnancy	0.23(0.19,0.29)	0.13(0.12,0.16)	0.012
Middle pregnancy	0.18(0.14,0.30)	0.16(0.12,0.20)	0.196
Late pregnancy	0.37(0.24,0.55)	0.16(0.12,0.21)	0.029
Before delivery	0.53(0.32,1.36)	0.18(0.16,0.26)	0.006

### Comparison of some laboratory indexes between two groups of pregnant women in late-pregnancy and before-delivery

The MAP, APTT, ALT, AST, Cr, UA, GGT, and GPR of pregnant women in the HELLP group showed an upward trend during the late pregnancy period and before-delivery period. They were all significantly higher than those of the control group, with statistically significant differences (*P* < 0.05). The PLT and FIB of pregnant women in the HELLP group showed a downward trend from late-pregnancy to before-delivery. They were significantly lower than those of the control group (*P* < 0.05). There was no significant difference in HBG and TBil levels between the two groups of pregnant women at before-delivery (*P* > 0.05) (Table 3). Spearman correlation analysis showed that GPR in late-pregnancy and before-delivery is correlated with MAP (*r* = 0.518,0.551, *P* < 0.001).

**Table 3:** Comparison of selected laboratory indexes in late-pregnancy and before-delivery between two groups of pregnant women

Variables	HELLP group (*n* = 50)	Control group (*n* = 50)	*P* value
MAP(mmHg)			
Early pregnancy	92.22 ± 10.74	83.02 ± 7.73	< 0.001
Middle pregnancy	92.42 ± 8.05	81.10 ± 5.88	< 0.001
Late pregnancy	102.54 ± 13.53	84.52 ± 5.83	< 0.001
Before delivery	117.38 ± 15.71	86.97 ± 6.42	< 0.001
HGB(g/dL)			
Early pregnancy	130.34 ± 8.33	126.53 ± 10.14	0.134
Middle pregnancy	121.63 ± 12.94	117.07 ± 9.73	0.062
Late pregnancy	126.81 ± 14.55	119.94 ± 9.87	0.007
Before delivery	122.60 ± 14.99	120.16 ± 10.75	0.352
FIB(g/L)			
Middle pregnancy	4.05 ± 0.82	4.11 ± 0.52	0.843
Late pregnancy	3.83 ± 1.30	4.58 ± 0.86	0.001
Before delivery	3.53 ± 1.05	4.60 ± 0.86	< 0.001
APTT(t/s)			
Middle pregnancy	26.26 ± 2.43	26.09 ± 2.37	0.874
Late pregnancy	27.02 ± 2.47	25.99 ± 1.99	0.029
Before delivery	27.12 ± 3.05	25.89 ± 1.66	0.014
ALT(U/L)			
Early pregnancy	34.57 ± 44.63	17.80 ± 15.68	0.083
Middle pregnancy	18.51 ± 20.56	20.63 ± 11.94	0.558
Late pregnancy	41.55 ± 45.40	12.48 ± 6.54	< 0.001
Before delivery	54.75 ± 49.25	11.66 ± 6.38	< 0.001
AST(U/L)			
Early pregnancy	21.34 ± 7.66	18.32 ± 5.90	0.161
Middle pregnancy	20.16 ± 9.19	21.99 ± 9.20	0.356
Late pregnancy	45.60 ± 45.63	17.55 ± 4.66	< 0.001
Before delivery	54.86 ± 50.42	16.86 ± 3.79	< 0.001
TBil (µmol/L)			
Early pregnancy	10.72 ± 4.78	11.00 ± 4.21	0.841
Middle pregnancy	7.32 ± 2.57	7.83 ± 2.78	0.396
Late pregnancy	11.12 ± 11.14	8.39 ± 2.90	0.097
Before delivery	10.29 ± 11.20	9.01 ± 4.11	0.454
Cr(µmol/L)			
Early pregnancy	49.09 ± 7.38	48.50 ± 5.20	0.761
Middle pregnancy	49.76 ± 9.49	46.44 ± 5.80	0.062
Late pregnancy	65.10 ± 21.13	51.31 ± 26.05	0.005
Before delivery	68.61 ± 21.09	47.48 ± 6.78	< 0.001
UA(µmol/L)			
Early pregnancy	230.02 ± 38.27	215.70 ± 38.95	0.271
Middle pregnancy	283.38 ± 75.22	253.51 ± 43.69	0.031
Late pregnancy	447.33 ± 129.58	290.52 ± 56.79	< 0.001
Before delivery	466.84 ± 146.71	304.07 ± 58.29	< 0.001

### Multivariate logistic regression analysis of risk factors for HELLP syndrome

Factors statistically significant in late pregnancy and before delivery were used as independent variables. After excluding the influence of collinearity, perform multivariate logistic regression analysis on late pregnancy laboratory indicators for predicting HELLP syndrome. The findings indicated that higher GPR, ALT, and UA were independent risk factors for the development of HELLP syndrome (OR = 23.382,1.169,1.016, *P* < 0.05), whereas higher FIB is a protective factor (OR = 0.057, *P* < 0.05). Compared with ALT, UA and FIB, the OR value of GPR is higher, which implies that the abnormal increase of GPR has better performance in predicting the onset of HELLP syndrome. The final formula for the multifactor binary logistic regression model was (Table 4)

$$\eqalign{& {\rm{In}}\left( {{\rm{P}}/1 - {\rm{P}}} \right){\rm{ }} \cr & = - 3.153 + 3.152*{\rm{GRP}} \cr & + 0.156*{\rm{ALT}} + 0.015*{\rm{UA}} \cr & - 2.867*{\rm{FIB}} \cr} $$

**Table 4:** Multivariate logistic regression analysis of risk factors for HELLP syndrome

Variables	B	SE	Wald X2	OR	95%CI	*P*
GPR	3.152	1.532	4.233	23.382	1.161 ~ 470.911	0.040
ALT	0.156	0.053	8.808	1.169	1.054 ~ 1.295	0.003
UA	0.015	0.005	9.629	1.016	1.006 ~ 1.025	0.002
FIB	-2.867	0.960	8.913	0.057	0.009 ~ 0.374	0.003
APTT	0.197	0.241	0.670	1.218	0.760 ~ 1.951	0.413

### Analysis of adverse pregnancy outcomes in two groups of pregnant women

Compared to the control group, the HELLP group had a considerably greater probability of preterm birth and MICU transmission, and the HELLP group had substantially more low birth weight babies than the control group (*P* < 0.05). There was no significant difference in the incidence of perinatal fetal distress and stillbirth between the two groups (*P* > 0.05) (Table 5). Spearman correlation analysis showed that in mid-pregnancy, GPR was not significantly associated with adverse pregnancy outcomes in HELLP syndrome (*P* > 0.05). However, elevated GPR levels in late pregnancy and before delivery were significantly correlated with preterm birth (*r* = 0.510, 0.450, *P* < 0.05). No significant correlation was found between GPR elevation and outcomes such as maternal ICU transfer, fetal distress, stillbirth, or low birth weight (*P* > 0.05). (Table 6).

**Table 5:** Comparison of adverse pregnancy outcomes between the two groups of pregnant women (*n* = 100)

Variables	HELLP group (*n* = 50)	Control group (*n* = 50)	*P* value
Preterm birth (n, %)	32(64%)	1(2%)	< 0.001
Transfer to MICU (n, %)	35(70%)	0(0)	< 0.001
Fetal distress (n, %)	2(4%)	1(2%)	0.558
Stillbirth (n, %)	3(6%)	0(0)	0.242
Low birth weight (n, %)	35(70%)	0(0)	< 0.001

**Table 6:** Correlation analysis of GPR and adverse pregnancy outcomes in HELLP syndrome (*n* = 50)

GPR	Preterm birth		MICU transmission		Fetal distress		Stillbirth		Low birth weight	
	*r*	*P*	*r*	*P*	*r*	*P*	*r*	*P*	*r*	*P*
Middle pregnancy	0.176	0.391	0.011	0.955	0.252	0.205	0.03	0.881	0.15	0.466
Late pregnancy	0.510	< 0.001	0.145	0.332	0.101	0.499	0.186	0.210	0.199	0.184
Before delivery	0.45	< 0.001	0.159	0.271	0.035	0.807	0.149	0.302	0.188	0.195

### ROC curve analysis to predict HELLP syndrome

**Fig. 3:**
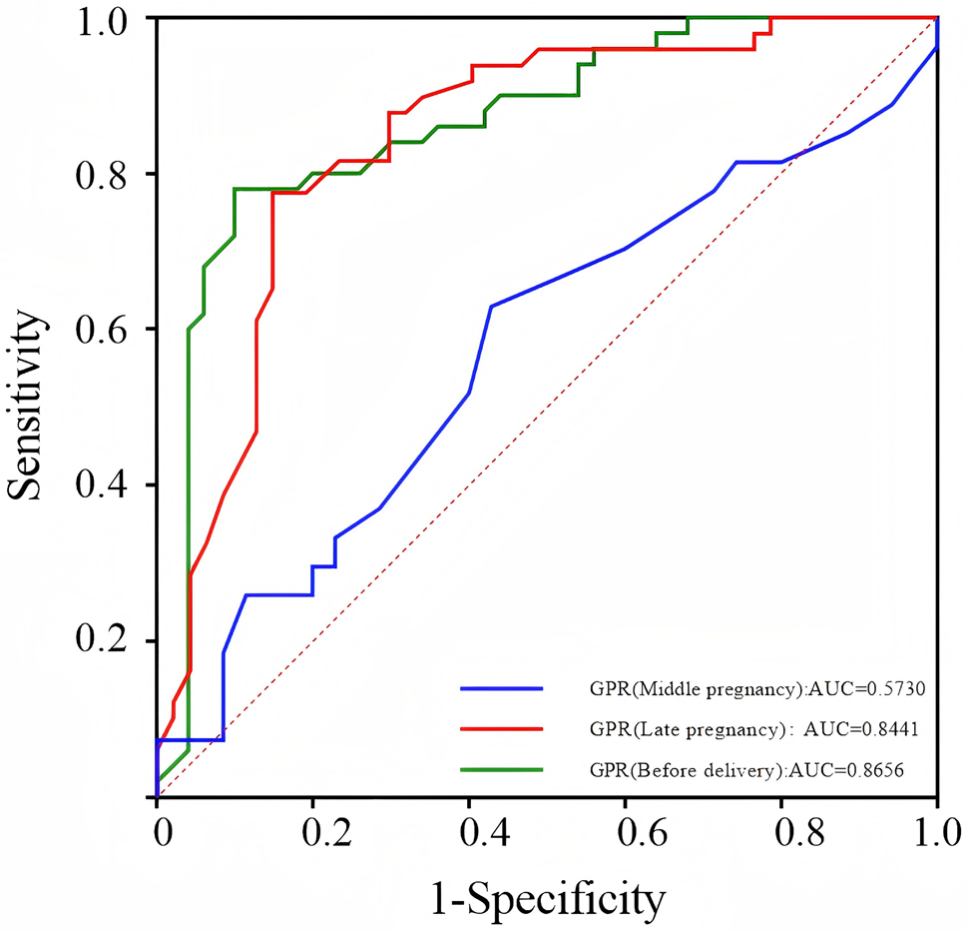
ROC curve for prediction of HELLP syndrome in mid-pregnancy、late-pregnancy and before delivery

**Fig. 4:**
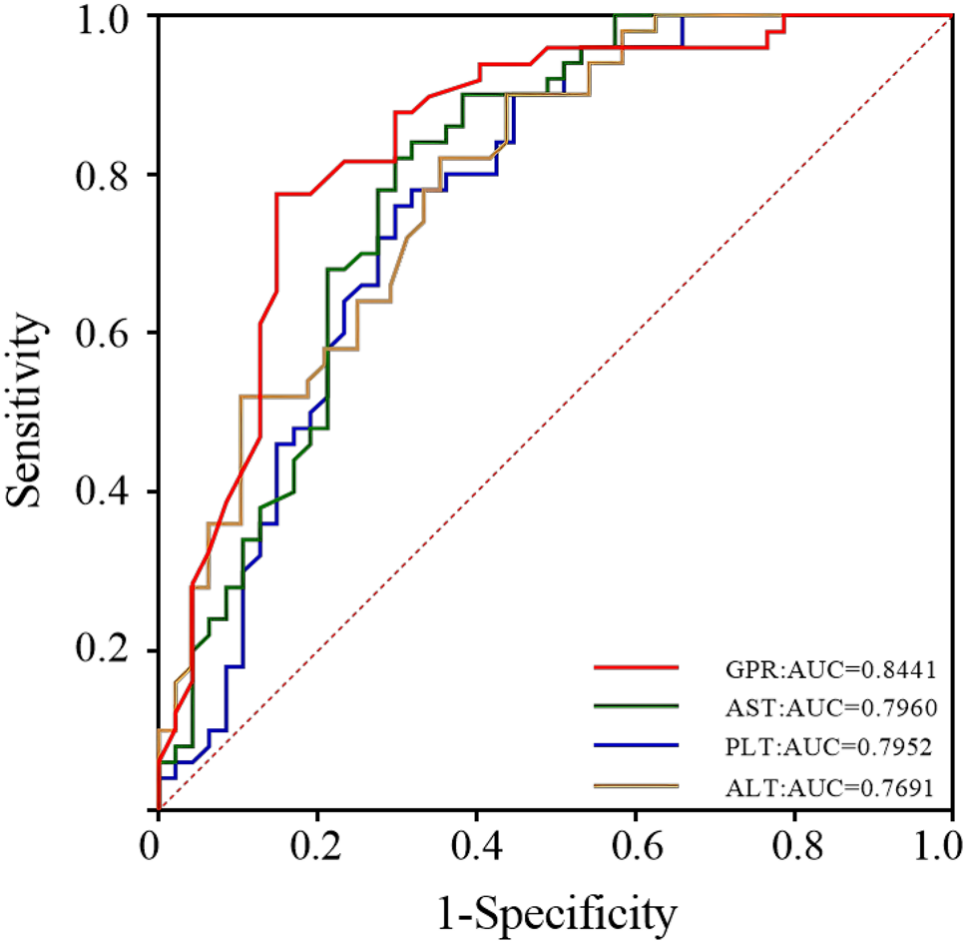
ROC curve for late-pregnancy HELLP syndrome prediction

Plot receiver operating characteristic (ROC) curves to assess the predictive efficacy of GPR for the onset of HELLP syndrome during mid-pregnancy, late pregnancy, and before delivery. The results showed that the area under the ROC curve (AUC) for GPR during mid-pregnancy was 0.5730 (95% CI: 0.4258 ~ 0.7202); the AUC for GPR during late pregnancy was 0.8441 (95% CI: 0.7627 ~ 0.9256); and the AUC for GPR before delivery was 0.8656 (95% CI: 0.7913 ~ 0.9399). In addition, the AUC for GPR during late pregnancy (0.8441) was higher than that of AST (AUC = 0.7960), PLT (AUC = 0.7952), and ALT (AUC = 0.7691) during the same period. HELLP syndrome sensitivity was estimated to be 77.6% and 78%, and specificity was 85% and 90%, respectively, when the GPR levels were 0.22 and 0.27. This suggests that GPR may have more excellent predictive value for HELLP syndrome than AST, ALT and PLT in late pregnancy, and that the sensitivity and specificity of GPR in predicting HELLP syndrome in before-delivery are higher than those in mid-pregnancy and late-pregnancy (Figs. 3 and 4).

## Discussion

The onset of HELLP syndrome is insidious, and the clinical manifestations are complex, diverse and lack of specificity, such as abdominal pain, nausea, vomiting, headache and visual impairment. The diagnosis of HELLP syndrome still mainly relies on laboratory indicators such as thrombocytopenia, elevated liver enzymes and hemolysis, which increases the risk misdiagnosis and missed diagnosis [7]. Thus, in order to better understand the progression of HELLP syndrome, early identification, precise diagnosis, and prompt clinical treatment are critical for improving maternal and infant adverse pregnancy outcomes.

The serum gamma-glutamyl transferase to platelet ratio (GPR) was first proposed by Lemoine et al. [4] to predict the risk of liver fibrosis and cirrhosis in patients with chronic hepatitis B. GPR is currently mainly used to assess liver-related diseases, such as liver fibrosis in chronic hepatitis B and non-alcoholic fatty liver disease, as well as to predict the development and prognosis of hepatocellular carcinoma [8–10]. However, research on the application of GPR in pregnancy-related hypertensive disorders is still limited. This study analyzed the dynamic changes of GGT, PLT, and their ratio (GPR) throughout pregnancy in women with HELLP syndrome. It was found that women with HELLP syndrome exhibited elevated GGT levels and decreased PLT levels throughout pregnancy. Moreover, the GPR showed a continuous increase, particularly in late pregnancy and before delivery, significantly higher than that of the control groups. Through multivariate logistic regression and ROC curve analysis, it was further confirmed that, in late pregnancy, GPR (OR = 23.382, AUC = 0.8441) outperformed ALT, AST, and PLT in predicting the onset of HELLP syndrome. Spearman correlation analysis indicated a positive correlation between GPR and MAP in late pregnancy and before delivery (*r* = 0.518, 0.551, *P* < 0.001), suggesting that a significant increase in GPR may be associated with the occurrence or worsening of hypertension. Finally, we also found that late pregnancy GPR may be associated with adverse pregnancy outcomes, such as preterm birth.

However, the pathogenesis of HELLP syndrome has not yet been fully elucidated. VAN et al. [11, 12] suggested that its pathological process is closely related to the inflammatory response of the placenta-endothelial-liver axis. Placental ischemia and hypoxia induce the release of anti-angiogenic factors, necrotic debris, and cell-free DNA into the bloodstream. These substances then reach the liver through the circulatory system, where they activate liver sinusoidal endothelial cells (LSECs), triggering a systemic inflammatory cascade response [12]. The damage to liver sinusoidal endothelial cells and the formation of microthrombi can disrupt the microcirculation of the liver sinusoids, ultimately leading to ischemic injury of hepatocytes. This increases the permeability of the cell membrane, facilitating the release of GGT from the cytoplasm into the bloodstream [13]. In addition, activated liver sinusoidal endothelial cells, under inflammatory stimulation, promote platelet activation, adhesion, and aggregation, leading to a consumptive decrease in platelets. Meanwhile, the formation of microthrombi within the sinusoidal spaces further accelerates platelet depletion [14]. Thus, GPR, by combining platelet consumption and the elevation of GGT, can more sensitively indicate ischemic injury in the liver sinusoidal spaces.

GGT is not only a sensitive indicator of liver damage, but it has also become an important biomarker for inflammation and early prediction of oxidative stress. Its application has expanded beyond liver-related conditions to include diseases involving ischemia-reperfusion injury [15–17]. Recent studies have shown that GGT levels in pregnant women with hypertension significantly increase as pregnancy progresses, while in healthy pregnancies, GGT tends to decrease with advancing gestational age. Moreover, elevated GGT levels are closely associated with the severity of the disease and adverse pregnancy outcomes [18, 19]. Currently, there is limited research on the relationship between GGT and HELLP syndrome. In this study, we found that GGT levels in women with HELLP syndrome steadily increased from mid-pregnancy to before delivery, with levels significantly higher than those in the control group during late pregnancy and before delivery. These findings further underscore the potential clinical value of GGT in predicting HELLP syndrome. A decrease in PLT is one of the essential criteria for diagnosing HELLP syndrome [20]. Rinehart and Chen et al. [21, 22] observed that the severity of HELLP syndrome in pregnant women is closely linked to platelet consumption. Furthermore, a significant decrease in platelet count was associated with a higher risk of adverse maternal and fetal outcomes. In this study, we also observed a continuous decrease in PLT in pregnant women with HELLP syndrome from early pregnancy to delivery, with values significantly lower than those in the control group throughout the pregnancy. These findings suggest that pregnant women with a sustained decline in PLT should be closely monitored for any changes in their condition, enabling early detection and prevention of the development of HELLP syndrome.

Recent advancements have been made in the development of biomarkers for predicting HELLP syndrome. However, their clinical application is still hindered by challenges related to cost and accessibility. Zhang and Lind et al. [23, 24] have identified several biomarkers, including hypoxia-inducible factor-1α (HIF-1α), placental growth factor (PLGF), pregnancy-associated plasma protein-A (PAPP-A), and soluble fms-like tyrosine kinase-1 (sFlt-1), which have shown predictive value for the development of HELLP syndrome in women with preeclampsia. Although these novel biomarkers exhibit high specificity, their sensitivity remains relatively limited. Additionally, factors such as high testing costs, complex detection processes, strict operational requirements, and limited standardization significantly hinder their widespread clinical application. Therefore, using routine laboratory indicators to predict HELLP syndrome offers high practicality and accessibility. It enables rapid and cost-effective assessment in most clinical settings, providing a viable alternative for the early identification and management of HELLP syndrome.

Studies have explored the use of inflammatory markers, such as the neutrophil-to-monocyte and lymphocyte count ratio (SIRI) and the eosinophil-to-monocyte ratio (EMR), to predict preeclampsia and HELLP syndrome [25, 26]. However, these indicators have been found to be ineffective in predicting the disease during early pregnancy, with their predictive value only emerging closer to delivery. In contrast, GPR, due to its unique pathophysiological basis, demonstrates greater potential for clinical application. GPR, by integrating liver injury (GGT) and coagulation dysfunction (PLT), provides a more comprehensive reflection of the multi-system damage mechanism in the placenta-endothelial-liver axis, demonstrating significant predictive efficacy, particularly in the late pregnancy period (AUC = 0.8441). Additionally, GPR is based on routine laboratory indicators, making it cost-effective and easily accessible, which makes it particularly suitable for widespread use and promotion in resource-limited areas. However, the limitation of GPR is that it may be influenced by non-pregnancy-related liver diseases, and its predictive effectiveness during early pregnancy still requires further validation. Future studies should explore the combined model of GPR with other indicators, such as uterine artery blood flow, to optimize its predictive value for HELLP syndrome and provide a more reliable basis for early intervention.

## Conclusion

In conclusion, the abnormally elevated GPR in late pregnancy has a certain predictive value for HELLP syndrome and its adverse pregnancy outcomes. Therefore, it is important to regularly monitor the changes in GPR of pregnant women during pregnancy to early identify HELLP syndrome, and take effectively preventive and intervention measures timely to improve pregnancy outcomes. However, this study is a retrospective, single-center study with relatively small number of cases and its outcomes may have limitations. To further validate the role of GPR in predicting HELLP syndrome, a larger-scale, multi-center prospective study should be conducted in the future, while collecting more pregnancy-related examination data to provide more robust scientific evidence for more accurate early-identification of HELLP syndrome and improvement of adverse pregnancy outcomes.

## Data Availability

The data in this study can be obtained from the corresponding author.
